# Lemierre Syndrome With Atypical Cardiological Involvement and Fatal Outcome

**DOI:** 10.7759/cureus.95424

**Published:** 2025-10-26

**Authors:** Aisha Jamal, Shamsuddin Mir Jan, Amara Zafar, Mustafa Jaffar, Taymmia Ejaz

**Affiliations:** 1 Medicine, Aga Khan University Hospital, Karachi, PAK; 2 Medicine, Army Medical College, Islamabad, PAK

**Keywords:** bacteroides, fusobacterium, lemierre, oropharyngeal infection, septic thrombophlebitis

## Abstract

Lemierre syndrome, an uncommon but potentially fatal condition, begins in the throat, usually in the area surrounding the tonsils or pharynx, spreading to other parts of the body. Septic thrombophlebitis, marked by the blood clots in veins due to bacterial infection, is the hallmark of Lemierre syndrome. We describe a case of a 35-year-old male patient who presented with a sore throat, hoarseness, and swelling on the right side of the face and neck for one week, followed by a sudden onset of shortness of breath for one day. His condition deteriorated rapidly, and he was intubated and moved to the Intensive Care Unit. There was no recorded fever or history of fever. A moderate pericardial effusion was discovered by bedside echocardiogram. A computed tomography (CT) scan showed bilateral consolidation, septic emboli, mediastinal empyema, pulmonary embolism, and thrombosed veins. A positive blood culture for *Bacteroides *species confirmed the diagnosis of Lemierre syndrome. The patient's health worsened even with vigorous treatment, and, unfortunately, he passed away. Clinicians should maintain a high level of suspicion for this disease in patients with bacterial infection symptoms, especially originating in the head and neck regions, as well as in patients presenting with swelling of the neck. This report aims to provide valuable insights into the complexities of Lemierre syndrome, prompting further research and awareness to enhance early detection and improve outcomes for such critical individuals.

## Introduction

Lemierre syndrome, also known as post-anginal sepsis, is an uncommon but serious illness that is caused by a bacterial infection that starts in the throat, particularly in the area of the tonsils or pharynx, and later spreads to the neck and other parts of the body. It was first described by André Lemierre in 1936 as a complication of tonsillitis, and since then, there have been several instances of Lemierre syndrome recorded in medical reports [1]. Septic thrombophlebitis, marked by the blood clots in veins due to bacterial infection, is the hallmark of Lemierre syndrome. These emboli can result in a range of serious complications, such as sepsis, lung abscesses, and mortality, if not urgently addressed [2]. It is a rare but life-threatening infection, more commonly reported in young males, with a ratio of 2:1, and has an overall prevalence of 0.6-3.6 cases per million, although the incidence continues to increase through the years [3]. Although the incidence of Lemierre syndrome is rare, it is important for medical practitioners to be aware of this condition and to consider it in patients presenting with fever, sore throat, and other symptoms of bacterial infection, leading to rapid dissemination. Here, we report a case of Lemierre syndrome in a 35-year-old male presenting with the complaint of sore throat, hoarseness, and swelling on the right side of the face and neck for one week, followed by the sudden onset of shortness of breath.

## Case presentation

A 35-year-old male patient with a history of substance abuse (cocaine, heroin, and tobacco) was admitted to the Intensive Care Unit (ICU) after presenting to the Emergency Department (ED), with a sore throat, hoarseness, and swelling on the right side of the face and neck for one week, followed by a sudden onset of shortness of breath for one day. His family reported low-grade fever, which was undocumented and subsided with antipyretics for one week. Before this incident, his family reported a normal functional status and no prior history of any comorbid conditions.

Upon arrival in the ED, the patient was tachypneic, with a respiratory rate of 50 breaths per minute, a heart rate of 150 beats per minute, blood pressure of 95/88 mmHg, and an oxygen saturation of 85% on room air. Chest auscultation revealed decreased air entry in the right lower lung zone. Examination of the precordium was unremarkable, with normal intensity of heart sounds and no audible murmur. There was swelling in the left upper limb, predominantly involving the left arm. Non-invasive ventilation (NIV) did not bring about any significant improvement. Eventually, he was intubated for invasive ventilation due to worsening respiratory distress and placed on assisted control/volume control (AC/VC) mode with a positive end-expiratory pressure (PEEP) of 5 cm H_2_O and a fraction of inspired oxygen (FiO_2_) of 80% and was shifted to the ICU.

Echocardiography revealed a moderate pericardial effusion. Pericardiocentesis was performed, draining 320 mL of hemorrhagic fluid, which was then sent for biochemistry, acid-fast bacilli staining, and microbiology cultures. The patient’s pericardial fluid analysis showed a protein level of 4,500 mg/dL. Complete blood count (CBC) showed leukocytosis of 25.9 x 10^9^/L, characterized by 90% neutrophils and 6.3% lymphocytes, along with elevated inflammatory markers, including a C-reactive protein (CRP) of 237 mg/dL and a procalcitonin level of 13.7 ng/mL. Liver function tests were within normal limits. Blood cultures, which take time to grow, later identified the presence of *Bacteroides *species (fragilis group) after the patient’s death. Pericardial fluid analysis for fungi, acid-fast bacilli, and Gram stain and culture sensitivity yielded negative results (Table 1).

**Table 1:** Laboratory investigations. KFT, kidney function test; BUN, blood urea nitrogen; Na, sodium; K, potassium; Cl, chloride; BIC, bicarbonate; CBC, complete blood count; Hb, hemoglobin; WBC, white blood cells; PT, prothrombin time; INR, international normalized ratio; APTT, activated partial thromboplastin time; plasma Trop- I, troponin Inhibitory protein; plasma Nt- ProBNP, N-terminal pro-B-type natriuretic peptide; CRP; C-reactive protein

Test	Observed Value	Reference Range		
KFT				
Blood urea nitrogen	11.8 mg/dL	7-20 mg/dL		
Serum creatinine	2.2 mg/dL	0.9-1.3 mg/dL		
Na	129 mmol/L	135-145 mmol/L		
BIC	8.5 mmol/L	20-31 mmol/L		
CL	89 mmol/L	98-107 mmol/L		
K	5.3 mmol/L	3.5-5.0 mmol/L		
CBC				
Hemoglobin	12.4 g/dL	12.1-16.6 g/dL		
WBC	25.9 x 10^9^/L	4.8-11.3 x 10^9^/L		
Platelets	577 x 10^9^/L	154-433 x 10^9^/L		
Coagulation profile				
PT	18 seconds	9.3-12.8 seconds		
INR	1.8	0.9-1.2		
APTT	27 seconds	25-35 seconds		
Fibrinogen	512 mg/dL	156-400 mg/dL		
Cardiac Markers				
Plasma Trop- I( High Sensitivity)	7 ng/L	0-57 ng/L		
Plasma NT- ProBNP	7206 Pg/mL	<125 pg/mL		
Infective Markers				
C-reactive protein	183.59 mg/L	0-10 mg/L		
Procalcitonin	4.60 ng/mL	<0.5 ng/mL		

**Figure 1:**
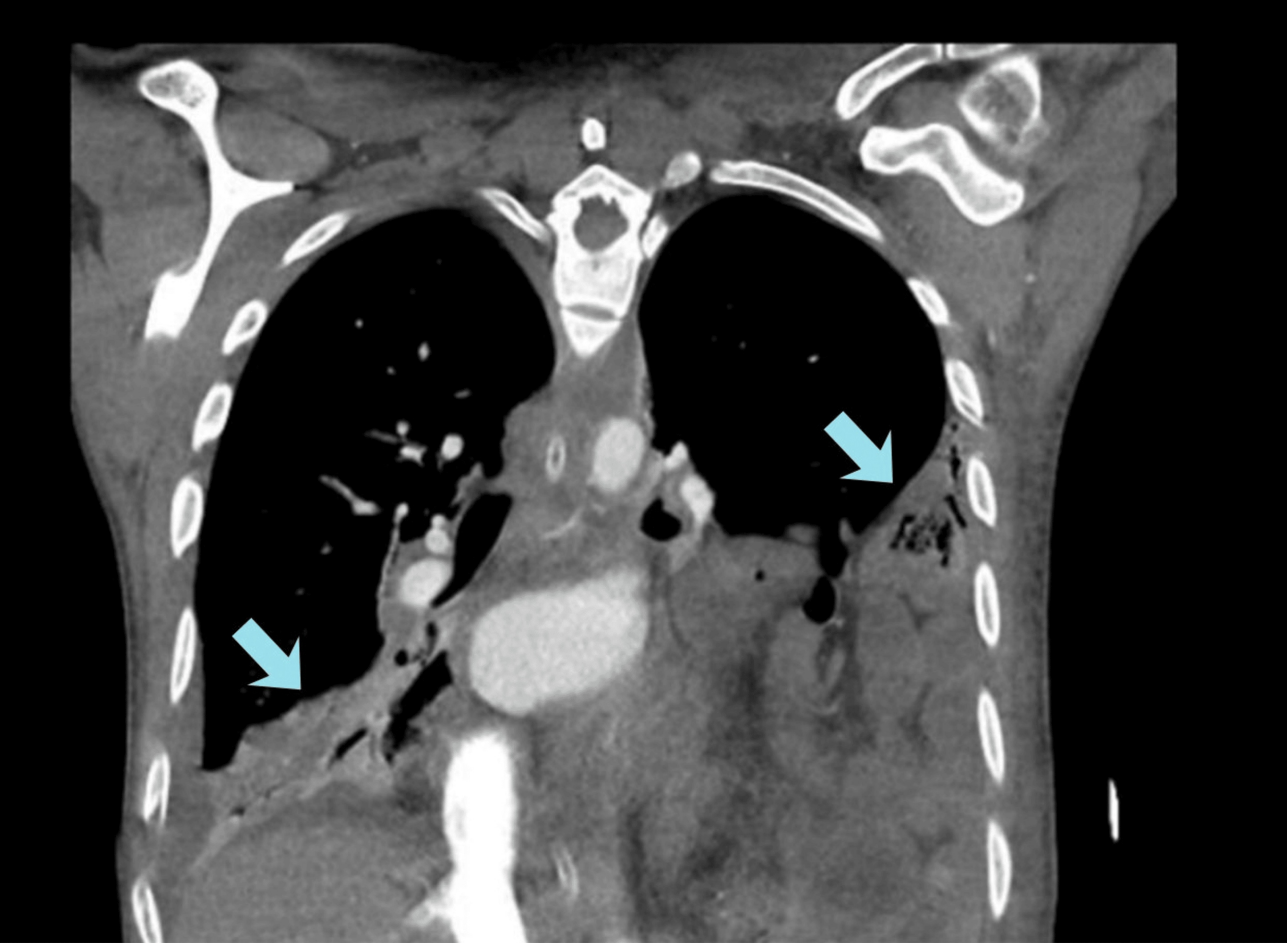
Computed tomography (CT) chest with intravenous contrast showing multiloculated high-density fluid collections formation, representing empyema, within the mediastinal region.

Computed tomography (CT) chest with intravenous contrast was done, which revealed bilateral consolidation with air bronchogram involving the left lower lobe and left lingular segment and the right middle and lower lobe with parapneumonic effusion suggestive of septic emboli (Figure 1).

It also showed multiloculated high-density fluid collection formation, representing empyema within the mediastinal region. Large high-density collection within the anterior mediastinum extending from the superior border of manubrium sterni to the body of sternum, measuring approximately 112 x 63 x 30 mm with an approximate volume of 105 mL, was also seen. Filling defects were identified in the subsegmental branches of the bilateral pulmonary artery vasculature, suggestive of pulmonary embolism. A thrombosed left internal jugular vein and an attenuated proximal part of the left subclavian vein and an incidental finding of left-sided diaphragmatic hernia were also seen. Since clinical, microbiological, and radiological findings correlated, a diagnosis of Lemierre syndrome was made.

He was hemodynamically unstable, requiring triple inotropic support, and had refractory shock. He was started on meropenem 1,000 mg every eight hours, vancomycin 1,000 mg every 12 hours, and metronidazole 500 mg every 8 hours empirically since he died within 24 hours of presentation, and cultures take time to grow organisms. He had multiple runs of supraventricular tachycardia (SVT) and ventricular tachycardia (VT) requiring multiple electrical cardioversions. Repeat review echocardiogram showed mild pericardial effusion. Despite triple inotropic support, adequate mean arterial pressure could not be maintained, and persistent runs of VT occurred. Antiarrhythmic agents were started, but VT persisted. The family was counselled in detail regarding the grave prognosis of the patient, and they decided on the withdrawal of ventilator support. After the code was changed to Do-Not-Resuscitate (DNR) comfort care, the patient was terminally extubated. Post extubation, he went into cardiac arrest and died within 15 hours of being shifted to the ICU.

## Discussion

Lemierre syndrome is a rare disease caused by sepsis secondary to anaerobic bacteria, leading to thrombophlebitis of the internal jugular vein [4]. We present a rare case of a young male who presented with acute dyspnea, hoarseness, and edema of the face and neck, which was diagnosed as a case of Lemierre syndrome.

Lemierre syndrome is usually preceded by oropharyngeal infection presenting as sore throat in 33% of cases, neck mass in 23% cases, and pain in the neck in 20% of cases, subsequently resulting in septicemia one to three weeks later, with 58% of the patients requiring ICU admission [5]. Our patient presented with similar symptoms. Our patient went into refractory shock with multiple runs of VT and SVT refractory to electrical cardioversion. To the best of our knowledge, this has not been reported before with Lemierre syndrome, especially in a young individual. A case report and literature review by Chen et al. highlighted 12 cases of *Klebsiella pneumonia-*associated Lemierre syndrome, with mortality in only two cases secondary to respiratory failure [6].

The diagnosis of Lemierre disease is usually made on clinical or radiological grounds. As proposed by Raiordan, the triad of Lemierre disease encompasses a recent upper respiratory tract infection, clinical evidence of metastatic lesions, and either isolation of *Fusobacterium *species in blood culture or thrombophlebitis of the internal jugular vein [2]. The isolation of *Bacteroides fragilis *in our case adds to its atypical nature, as *Fusobacterium necrophorum *and *Fusobacterium nucleatum *are the organisms most commonly associated with Lemierre syndrome. A recent individual patient-level analysis of reported Lemierre syndrome cases found that 61% of microbiological specimens were positive for *Fusobacterium *species, while *Streptococcus *and *Staphylococcus *species accounted for 20%. Other organisms, including *Bacteroides*, were identified in only 6% of cases, highlighting the rarity of this finding [7].

Our patient had a prior history of intravenous (IV) drug abuse, including heroin and cocaine. Needle contamination could have been a possible source of infection and sepsis in our case. Fernadez et al reported a case of a 29-year-old Dominican man with a history of IV drug abuse diagnosed with Lemierre syndrome. Although contrary to our case, there was an absence of oropharyngeal abscess or other ear, nose, and throat symptoms [8]. Another case was reported by Lin et al. of a 64-year-old IV heroin user with thrombophlebitis and *P. aeruginosa *bacteremia, epidural abscess with spinal cord compression, and right jugular vein thrombosis [9]. A similar case report by Kidambi et al. discussed the association of IV drug abuse in a 24-year-old woman leading to methicillin-resistant *Staphylococcus aureus *(MRSA) bacteremia and Lemierre syndrome [10].

Internal jugular vein thrombosis can be detected on imaging in 30-70% of patients with Lemierre syndrome, extending into the subclavian, axillary, and brachiocephalic veins, as well as into the cerebral venous sinuses [11]. This was seen in our patient as well. Septic emboli as a result of thrombus extension and resulting sepsis is the most dreaded complication, with lung and pleura being the most common sites of metastatic spread, accounting for 97% cases, followed by the joints, muscles, bones, the liver, the skin, the spleen, and the endocardium [8-12]. Commonly identified complications of the disease, in addition to septic, pulmonary emboli include septic arthritis, osteomyelitis, cerebral abscess, meningitis, epiduritis, vertebral thrombosis with spondylitis, and disseminated intravascular coagulation (DIC) [13].

Pericarditis with pericardial effusion and a significant arrhythmia were observed in our patient - both rare manifestations of Lemierre syndrome. Cardiac involvement has been reported in fewer than 10% of cases, and arrhythmias as severe as the one seen here have not been previously described [14]. Furthermore, this disease, which is often seen in adolescents, can be seen in the elderly. A recent case reported from Peshawar, Pakistan, involved a 67-year-old woman with a travel history, presenting with fever, cough, sore throat, shortness of breath, and discharging cervical sinuses. This was later diagnosed as Lemierre syndrome, which was recognized early and successfully treated with antibiotics [15].

Doppler ultrasound is a highly specific modality to identify thrombus, but owing to its low sensitivity, a CT scan remains the investigation of choice in the diagnosis of internal jugular vein thrombosis, abscesses and septic emboli, and distant metastatic lesions associated with Lemierre syndrome [14].

Antibiotics remain the main treatment modality. A systematic review by Moretti et al. showed that the use of amoxicillin/clavulanic acid and ceftriaxone was the preferred drug over metronidazole [16]. Although the use of anticoagulation has been controversial, some authors have advocated the role of anticoagulation, with duration varying between weeks to months depending on the severity, in certain instances, including no response to antibiotic therapy after 48-72 hours, persistent bacteremia, pre-existing thrombophilia, and progression of intracranial thrombosis [17,18]. The case serves as a reminder that, while fatality from the disease has decreased considerably since the introduction of antibiotics and is considered to be lower than 5%, Lemierre is still a potentially fatal disease [19].

## Conclusions

Lemierre syndrome is a rare but severe condition that can lead to life-threatening complications if not identified and treated promptly. Clinicians should maintain a high level of suspicion for this disease in patients with bacterial infection symptoms, especially originating in the head and neck regions, and patients presenting with swelling of the neck. Early treatment initiation can have a positive impact on patient outcomes and mitigate the potential for severe complications. This case serves as an excellent reminder of the typical clinical features of Lemierre syndrome-persistent fever and chest or neck symptoms following pharyngitis in a previously healthy young adult - with septic pulmonary emboli seen on CT imaging. As such, it is especially relevant for clinicians working in primary or emergency care. In addition, the case highlights an atypical but important aspect: the isolation of *B. fragilis *instead of the more commonly associated *F. necrophorum*, and the need to recognize that the microbiological spectrum of Lemierre syndrome may be broader than traditionally thought. Consequently, diagnosis should remain primarily clinical rather than solely microbiological, as various pathogens can cause this same clinical entity.
